# IL-10 and TGF-β1 may weaken the efficacy of preoperative anti-tuberculosis therapy in older patients with spinal tuberculosis

**DOI:** 10.3389/fcimb.2024.1361326

**Published:** 2024-03-20

**Authors:** Shanshan Li, Runrui Wu, Mengru Feng, Hong Zhang, Dongxu Liu, Fenghua Wang, Wen Chen

**Affiliations:** Department of Pathology, The Eighth Medical Center, General Hospital of the Chinese People’s Liberation Army, Beijing, China

**Keywords:** spinal tuberculosis, *Mycobacterium tuberculosis*, abscesses, IL-10, TGF-β1

## Abstract

Spinal tuberculosis is a common extrapulmonary type that is often secondary to pulmonary or systemic infections. *Mycobacterium tuberculosis* infection often leads to the balance of immune control and bacterial persistence. In this study, 64 patients were enrolled and the clinicopathological and immunological characteristics of different age groups were analyzed. Anatomically, spinal tuberculosis in each group mostly occurred in the thoracic and lumbar vertebrae. Imaging before preoperative anti-tuberculosis therapy showed that the proportion of abscesses in the older group was significantly lower than that in the younger and middle-aged groups. However, pathological examination of surgical specimens showed that the proportion of abscesses in the older group was significantly higher than that in the other groups, and there was no difference in the granulomatous inflammation, caseous necrosis, inflammatory necrosis, acute inflammation, exudation, granulation tissue formation, and fibrous tissue hyperplasia. B cell number was significantly lower in the middle-aged and older groups compared to the younger group, while the number of T cells, CD4^+^ T cells, CD8^+^ T cells, macrophages, lymphocytes, plasma cells, and NK cells did not differ. Meaningfully, we found that the proportion of IL-10 high expression and TGF-β1 positive in the older group was significantly higher than that in the younger group. TNF-α, CD66b, IFN-γ, and IL-6 expressions were not different among the three groups. In conclusion, there are some differences in imaging, pathological, and immune features of spinal tuberculosis in different age groups. The high expression of IL-10 and TGF-β1 in older patients may weaken their anti-tuberculosis immunity and treatment effectiveness.

## 1 Introduction

Tuberculosis (TB) is an infectious disease caused by *Mycobacterium tuberculosis* (Goossens et al., 2020), and the lungs are the most common site of infection (Guo et al., 2022). Spinal tuberculosis is often secondary to pulmonary infection, with the thoracolumbar spine being the most commonly affected area (Kubihal et al., 2022; Na et al., 2023). Early symptoms of spinal tuberculosis are often subtle and nonspecific, including back pain, muscle spasms, and stiffness. As the disease progresses, the patient experiences worsening symptoms, including abnormal spine curvature, abscesses, and limited mobility. In addition, spinal tuberculosis can cause weakness, numbness, and paralysis of the legs by compressing and damaging nerves. “Early, Regular, Complete, Combined, Moderate” is the principle of TB treatment. For patients with stable, non-damaged vertebral bone, a cure can be achieved through treatment with anti-tuberculosis drugs alone. Surgery is necessary when medical treatment fails, there is significant bone destruction or spinal instability, or when there is compression of the spinal nerves. Preoperative anti-tuberculosis treatment and other adjuvant therapies are required to minimize the risk of surgery and recurrence of TB. However, there is currently no standardized presurgical anti-tuberculosis treatment specification, and the choice of surgical timing may require a precise treatment plan tailored to the individual patient’s condition.

In most cases of spinal tuberculosis, imaging reveals erosion and bone destruction of multiple neighboring vertebrae, which results in degenerative changes in the disc. Some cases may be characterized by swelling in the paravertebral or psoas major muscles, as well as abscess formation (Rajasekaran et al., 2018). Due to the severity of the disease, treatment for spinal tuberculosis typically lasts over 9 months (Garg and Goyal, 2020). Pathological diagnosis of spinal tuberculosis involves H&E staining, acid-fast staining, and molecular biology testing, which are considered the gold standard. The typical pathological changes caused by TB are characterized by chronic granulomatous inflammation accompanied by caseous necrosis. We conducted a preliminary comparison of the pathologic features of pulmonary and spinal tuberculosis. Our findings showed that the proportion of caseous necrosis, acute inflammation, abscess, exudation, and granulation tissue formation was significantly higher in the spinal tuberculosis group (Wu et al., 2023).

The immune response is crucial in preventing *Mycobacterium tuberculosis* infection. The process involves three pathological changes: exudation, proliferation, and degeneration/necrosis(Wang L et al., 2020). Macrophages, lymphocytes, neutrophils, and inflammatory factors secreted by them form tuberculosis granulomas that limit the spread of *Mycobacterium tuberculosis* and kill it (Ginsberg et al., 2016). After an early stage of inadequate control, *Mycobacterium tuberculosis* infection often reaches a state of equilibrium characterized by immune control and bacterial persistence (Chandra et al., 2022). It has been discovered that inflammation-associated cytokines play a crucial role in the progression of inflammation while *Mycobacterium tuberculosis* infects the host (Ma et al., 2019). Interleukin-10 (IL-10) plays a role in reducing the inflammatory response by interacting with other inflammatory factors, thereby impacting the progression of spinal tuberculosis (Liu et al., 2014). The high expression of tumor necrosis factor-α (TNF-α) and interferon-γ (IFN-γ) in spinal tuberculosis lesions was found to be associated with protective immune cells, and an imbalance in the expression of TNF-α, IFN-γ, and transforming growth factor-β (TGF-β) may exacerbate spinal tuberculosis hypersensitivity (Chen et al., 2014). In addition, macrophage migration inhibitory factor (MIF) has chemokine-like functions and plays a key role in a variety of acute and chronic inflammatory diseases (Wang et al., 2019). It has been reported that MIF may play an important role in the occurrence, development, and damage of spinal tuberculosis in the population of the northern province of China (Wang J et al., 2020).

Patient immunity may play a role in determining the length of anti-tuberculosis treatment and the timing of surgery for spinal tuberculosis. For this research, we gathered patients who suffered from spinal tuberculosis at different ages. We compared the differences in clinicopathologic features, immune cell subtypes and numbers, and expression of inflammation-associated factors. We aimed to provide important reference data for the clinical timing of surgery and the planning of postoperative treatment programs.

## 2 Materials and methods

### 2.1 Specimen collection

All cases were collected between January 2017 and December 2022 from inpatients at the 8th Medical Center of PLA General Hospital. Criteria for inclusion: patients aged 18 years or older who had spinal surgery, a clinical and pathological diagnosis of *Mycobacterium tuberculosis* infection, and no anti-tuberculosis treatment before hospitalization. Exclusion criteria include pulmonary tuberculosis, autoimmune diseases, malignancies, hepatitis, HIV infection, severe fungal and bacterial infections, as well as severe hematologic disorders. The criteria for spinal surgery in patients is as follows: If the spinal tuberculosis lesion produces pus, tuberculosis granulation tissue, caseous necrotic material, or dead bone compressing the spinal cord leading to sensory-motor deficits, or if the tuberculosis lesion leads to disruption of the local stability of the spine or even the development of localized kyphosis and deformity, then surgery may be necessary.

This study included 64 cases of spinal tuberculosis. The patients were classified into different age groups: younger (18 to 39 years), middle-aged (40 to 59 years), and older (60 years and above).

### 2.2 H&E staining

All tissue samples were fixed in 4% paraformaldehyde, embedded in paraffin after gradient dehydration, sectioned into 3 µm thickness, and mounted on slides. The slides were then baked at 72°C for 30 min, dewaxed using xylene, washed sequentially with gradient ethanol, and stained with hematoxylin dye for 30 s. The following steps were carried out: differentiation with hydrochloric acid, blue reversion with ammonia, eosin dye application for 5 s, dehydration with gradient ethanol, transparency with xylene, and sealing with neutral resin. The basic histological features from H&E-stained sections were observed under a microscope.

### 2.3 Acid-fast staining

Sections of 3 µm thickness were taken from each sample and attached to slides. These slides were then baked at 72°C for 30 min, dewaxed with xylene, washed with ethanol, and stained with red carbolic acid dye solution for 2 h. After that, the slides were decolorized with hydrochloric acid until they turned light pink. The next steps included hematoxylin application for 10 s, differentiation with hydrochloric acid, blue reversion with ammonia, dehydration with gradient ethanol, transparency with xylene, and sealing with neutral resin.

### 2.4 Identification of mycobacterium

**Figure 1:**
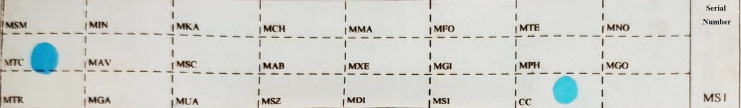
Sequence of probes on membrane strips. MTC: *Mycobacterium tuberculosis* complex; CC: quality control loci; The rest are non-*Mycobacterium tuberculosis* detection loci.

Ten sections of tissue specimens, each with a thickness of 5-10 µm, were taken to extract DNA. The process involved dewaxing, lysis, and enzymatic digestion of the samples. The DNA was added to PCR tubes from the *Mycobacterium* species identification gene test kit (Ya Neng Biotechnology Co., Ltd., Shenzhen, China) for amplification. The amplification products and membrane strips were combined in a tube with liquid A (890 mL purified water + 100 mL 20×saline sodium citrate + 10 mL 10% sodium dodecyl sulfate), heated in a boiling water bath for 10 min, and then hybridized at 59 °C for 1.5 h. After that, the membrane strips were submerged in Liquid B (965 mL purified water + 25 mL 20×saline sodium citrate + 10 mL 10% sodium dodecyl sulfate) at 59°C and washed for 15 min. The strips were then immersed in Liquid A [POD (peroxidase): Liquid A = 1:2000] for 30 min, followed by a 10-minute incubation in the color development solution [19 mL of Liquid C (900 mL purified water + 100 mL 1M sodium citrate) + 1 mL of TMB (3,3′,5,5′-Tetramethylbenzidine) + 10 µL of 3% hydrogen peroxide]. The reaction was halted using distilled water. The membrane strip would display blue spots if any were detected. The experiment was set up with a positive and negative control synchronized to ensure accurate results. (**Figure 1**).

### 2.5 Immunohistochemical staining

Sections of 3 µm thickness were taken from each sample and attached to slides. These slides were then baked at 72°C for 30 min and placed in an immunohistochemistry staining machine(Benchmark XT, Ventana Medical Systems Inc, Tucson, USA). Run the staining program, during which a primary antibody was added manually. After washing, the slides underwent hematoxylin staining for 10 seconds, hydrochloric acid differentiation, ammonia reversion to blue, gradient ethanol dehydration, xylene transparency, and neutral resin sealing. The antibodies used were as follows: CD3(RabMAb, ZA-0503), CD4(Murine monoclonal antibody, ZM-0418), CD8(RabMAb, ZA-0508), CD20(Murine monoclonal antibody, ZM-0039RUO), CD38(Murine monoclonal antibody, ZM-0422), CD56(Murine monoclonal antibody, ZM-0057), CD68(Murine monoclonal antibody, ZM-0060), LCA(Murine monoclonal antibody, ZM-0183). The above antibodies were purchased from OriGene China; TGF-β1 (RabMAb, ab215715), TNF-α(RabPAb, ab307164), IL-10(RabPAb, ab217941), IL-1β(RabPAb, ab283818), CD66b(RabPAb, ab214175) were purchased from Abcam(Shanghai)Trading Co. LTD; IL-6(RabPAb, NBP2-16957)was purchased from Novus Bio-Techne China Co., LTD; IFN-γ (RabMAb, MA5-42466) was purchased from Thermo Fisher Technology (China) Co., LTD; MIF (RabMAb, 87501S) was purchased from Cell Signaling Technology, INC. For the experiment, each slide was attached to a tissue wax slice showing positive expression of the corresponding protein. These wax slices were stained together with the sample tissue wax slices. Additionally, a negative control was set up for staining in each experiment.

### 2.6 Staining results judgment

The binding between antibodies and antigens is highly specific, and immunohistochemistry takes advantage of this principle to localize, characterize, and relatively quantify antigens (peptides and proteins) in tissues and cells. The presence of specific yellow or brown particles is considered to be positive. CD3, CD56, IL-1β (Interleukin-1β), TNF-α (Tumor necrosis factor-α), IL-6 (Interleukin-6) are positive in cytoplasm/membrane; CD4, CD8, CD20, CD38, LCA (Leukocyte common antigen), CD66b are positive in cell membrane; CD68, IL-10 (Interleukin-10), IFN-γ (Interferon-γ), TGF-β1 (Transforming growth factor-β1), MIF (Macrophage migration inhibitory factor) are positive in cytoplasm. Positive cell counts were determined for CD3, CD56, CD4, CD8, CD20, CD38, LCA, and CD68. Staining intensity of IL-1β, TNF-α, IL-6, CD66b, IL-10, IFN-γ, TGF-β1, and MIF was interpreted using “-”, “+”, “2+”, and “3+” results. Interpretation based on microscopic observation of staining results. “-” means negative, protein is not colored. “+” means weak positive. This indicates that the protein being tested is expressed at a low level, and the staining observed under the microscope appears yellow. “2+” and “3+” indicate strong positivity, with the protein being highly expressed, and the staining observed under the microscope appears brown or tan. Two pathologists with extensive experience in seeing tens of thousands of slides per year independently interpreted the experimental slides.

### 2.7 Statistical analysis

Photoshop CS6 software (Adobe Systems Incorporated, San Jose, USA) was applied to process the images, and SPSS Statistics 20.0 software (International Business Machines Corporation, Amunk, USA) was utilized for data analysis. Measurement data were presented in terms of the number of cases (n) and the corresponding percentage (%). The variables that follow normal distribution were represented by *_x*±s, and those not meeting normal distribution were expressed by P_50_ (IQR), and a non-parametric test was performed. Case numbers between groups were compared using chi-square or Fisher’s exact probability test. Statistically significant differences were represented as *P* < 0.05.

## 3 Results

### 3.1 Clinical data

The younger group comprised 20 patients, 11 males and 9 females, with a mean age of 26.70 years. The middle-aged group consisted of 7 men and 14 women, with an average age of 48.95 years. The older group consisted of 23 patients, 6 males and 17 females, with an average age of 67.61 years. In all three groups, the main pathogenic site was the thoracic and lumbar spine. There was no statistical difference between the groups in terms of general information, such as gender and pathogenic site.

**Figure 2:**
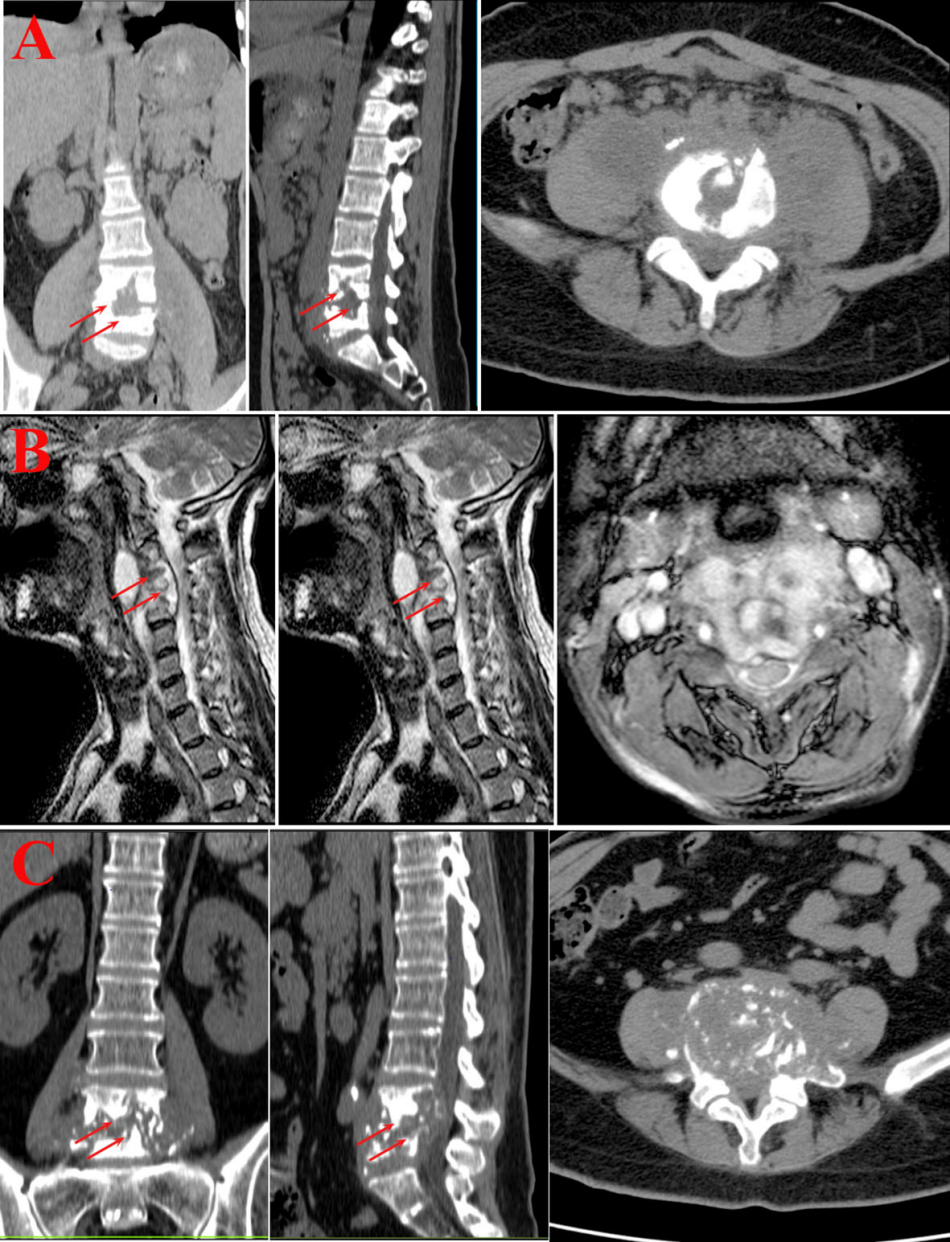
Imaging examination. **(A)** Younger group. Bone destruction of the lumbar vertebrae with narrowing of the corresponding intervertebral space, swelling of the paravertebral soft tissues and bilateral lumbar psoas muscles, and the formation of doughnut-shaped abscesses. **(B)** Middle-aged group. The cervical vertebral body shows varying degrees of bone destruction, with accompanying significant narrowing and disappearance of the intervertebral space. The adjacent soft tissues at the corresponding level display swelling and formation of abscesses. **(C)** Older group. Bone destruction of lumbar vertebrae, narrowing and disappearance of the corresponding intervertebral space, swelling of paravertebral soft tissues and bilateral lumbar psoas muscles, and formation of doughnut-shaped abscesses.

No difference in bone destruction or paravertebral soft tissue edema was found among the three groups in imaging features. The percentage of bilateral psoas major edema was significantly greater in the younger group compared to the older group. On the other hand, the percentage of abscess formation was significantly lower in the older group compared to the younger and middle-aged groups. Furthermore, the percentage of osteoproliferation was significantly higher in the older group compared to the other two groups. (**Table 1**, **Figure 2**).

**Table 1:** Comparison of clinical data and imaging features.

	Younger (n=20)	Middle-aged (n=21)	Older (n=23)	*P*
Gender (Male)	55% (n=11)	33.33% (n=7)	26.09% (n=6)	0.132
Age (year)	26.70 ± 5.33	48.95 ± 5.38	67.61 ± 5.36	0.000
TB Type (MTB)	100% (n=20)	100% (n=21)	100% (n=23)	/
Methods				
Acid-fast (+)	50% (n=10)	61.90% (n=13)	39.13% (n=9)	0.320
PCR (+)	100% (n=20)	100% (n=21)	100% (n=23)	/
spinal site				
Cervical	0 (n=0)	9.52% (n=2)	4.35% (n=1)	0.519
Thoracic	25% (n=5)	33.33% (n=7)	56.52% (n=13)	0.086
Lumbar	60% (n=12)	57.14% (n=12)	34.78% (n=8)	0.186
Thoracolumbar	10% (n=2)	0 (n=0)	4.35% (n=1)	0.403
Lumbosacral	5% (n=1)	0 (n=0)	0 (n=0)	0.312
Imaging Characteristics				
Destruction of bone	95% (n=19)	95.24% (n=20)	91.30% (n=21)	1.000
Paravertebral soft tissue edema	80% (n=16)	76.19% (n=16)	91.30% (n=21)	0.383
Bilateral psoas major muscle edema	40% (n=8)^*^	14.29% (n=3)	8.70% (n=2)	0.036
Abscess formation	65% (n=13)^*^	71.43% (n=15)^#^	34.78% (n=8)	0.032
Osteoproliferation	15% (n=3)^*^	71.43% (n=15)^#^	95.65% (n=22)	0.000
Intervertebral changes	100% (n=20)	95.24% (n=20)	78.26% (n=18)	0.042

Please note that an asterisk (*) indicates a statistically significant difference between the younger and older groups, and a pound sign (#) indicates a statistically significant difference between the middle-aged and older groups.

### 3.2 Pathological features

**Figure 3:**
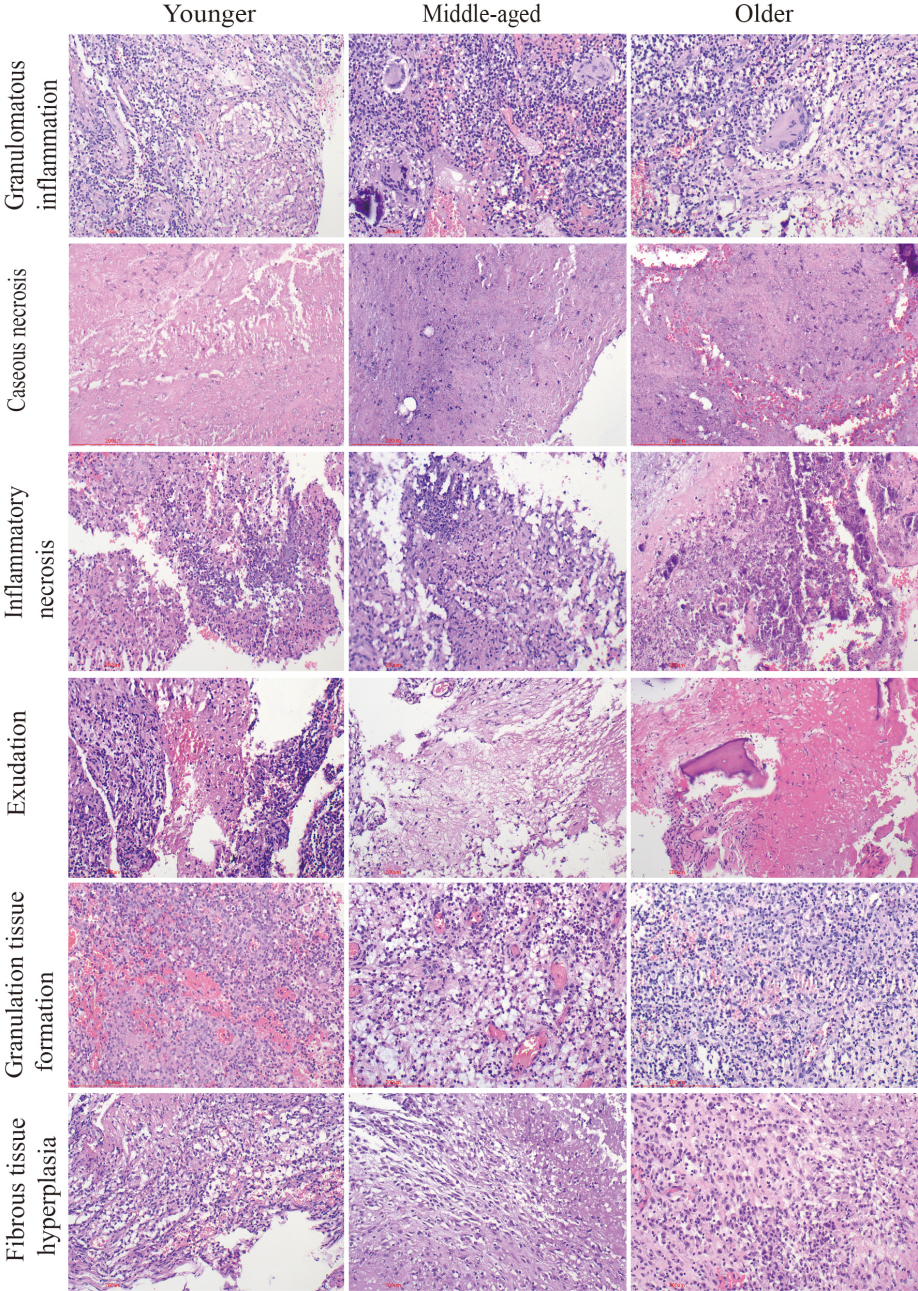
Pathological characteristics of the three groups. Chronic granulomatous inflammation with caseous necrosis is a typical pathologic change in spinal tuberculosis. H&E staining revealed inflammatory necrosis, exudation, the formation of granulation tissue, and fibrous tissue proliferation. Caseous necrosis is a type of coagulative necrosis characterized by the presence of unstructured red-stained granular material. Tissue necrosis caused by inflammation is known as inflammatory necrosis. Scale bar: 200 μm; Image magnification: H&E (200×).

The typical pathological feature of spinal tuberculosis in all groups was chronic granulomatous inflammation with caseous necrosis. In addition, inflammatory necrosis, exudation, acute inflammation, granulation tissue formation, and fibrous tissue hyperplasia were observed. It was found that there were no significant differences between the three groups in terms of various pathological features such as granulomatous inflammation, caseous necrosis, inflammatory necrosis, exudation, acute inflammation, granulation tissue formation, and fibrous tissue hyperplasia. It is interesting to note that the proportion of abscesses was significantly higher in the older group as compared to the younger and middle-aged groups, which is opposite to the imaging presentation. (**Table 2**, **Figure 3**).

**Table 2:** Comparison of pathological features.

Pathology	Younger (n=20)	Middle-aged (n=21)	Older (n=23)	*P*
Granulomatous inflammation	85% (n=17)	76.19% (n=16)	69.57% (n=16)	0.534
Caseous necrosis	100% (n=20)	80.95% (n=17)	82.61% (n=19)	0.114
Abscess	30% (n=6)^*^	33.33% (n=7)^#^	65.22% (n=15)	0.034
Inflammatory necrosis	50% (n=10)	52.38% (n=11)	73.91% (n=17)	0.205
Exudation	65% (n=13)	57.14% (n=12)	69.57% (n=16)	0.688
Acute inflammation	85% (n=17)	95.24% (n=20)	91.30% (n=21)	0.582
Granulation tissue formation	65% (n=13)	85.71% (n=18)	78.26% (n=18)	0.325
Fibrous tissue hyperplasia	70% (n=14)	61.90% (n=13)	60.87% (n=14)	0.798

Please note that an asterisk (*) indicates a statistically significant difference between the younger and older groups, and a pound sign (#) indicates a statistically significant difference between the middle-aged and older groups.

### 3.3 Immune cell infiltration

**Figure 4:**
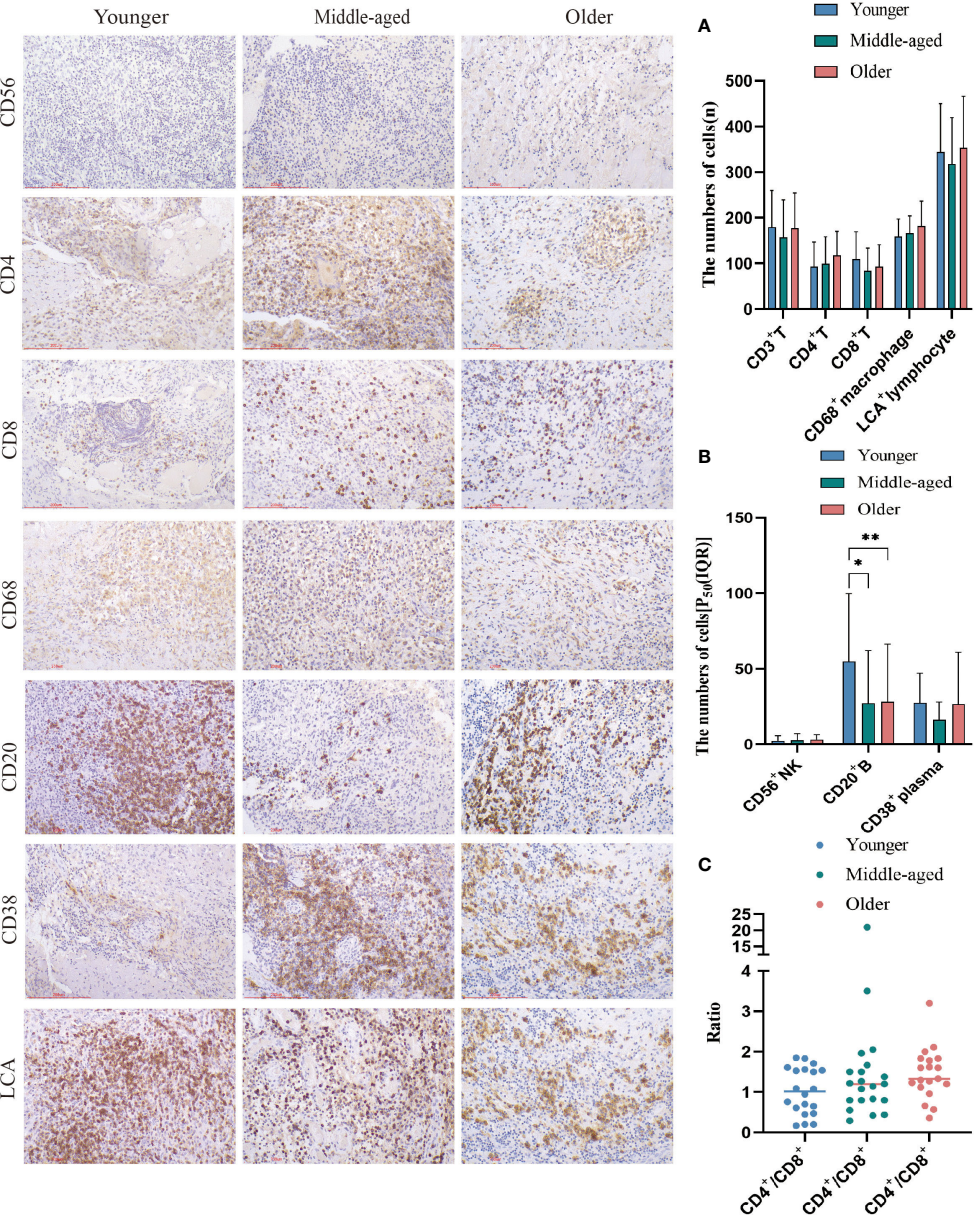
Immune cell infiltration and statistical analysis of the three groups. Immunohistochemical staining was used to label immune cell (CD20^+^B cells, CD4^+^T cells, CD68^+^ macrophages, LCA^+^ lymphocytes, CD38^+^plasma cells, CD56^+^NK cells and CD8^+^T cells) infiltration. **(A, B)** Statistical analysis of the number of immune cells. **(C)** Analysis of the ratio of CD4^+^ to CD8^+^. Scale bar: 200 μm; Image magnification: immunohistochemical staining (200×). Please note that an asterisk (*) indicates a significant difference and two asterisks (**) indicate an extremely significant difference.

In spinal tuberculosis, the predominant infiltrating immune cells are macrophages. B cells and plasma cells were mostly focally infiltrated. Extensive infiltration of CD4^+^T cells and CD8^+^T cells was observed, while the number of infiltrating NK cells was relatively low. The statistical analysis showed that there were no significant differences in the numbers of T cells, CD4^+^T cells, CD8^+^T cells, CD68^+^ macrophages, LCA^+^ lymphocytes, CD38^+^ plasma cells, and CD56^+^ NK cells among the three groups. However, the younger group had a significantly higher number of CD20^+^ B cells than the middle-aged and older groups, but its clinical relevance remains unclear. The CD4^+^/CD8^+^ ratio was also not statistically different among the three groups. (**Figure 4**).

### 3.4 Expression of inflammatory factors

**Figure 5:**
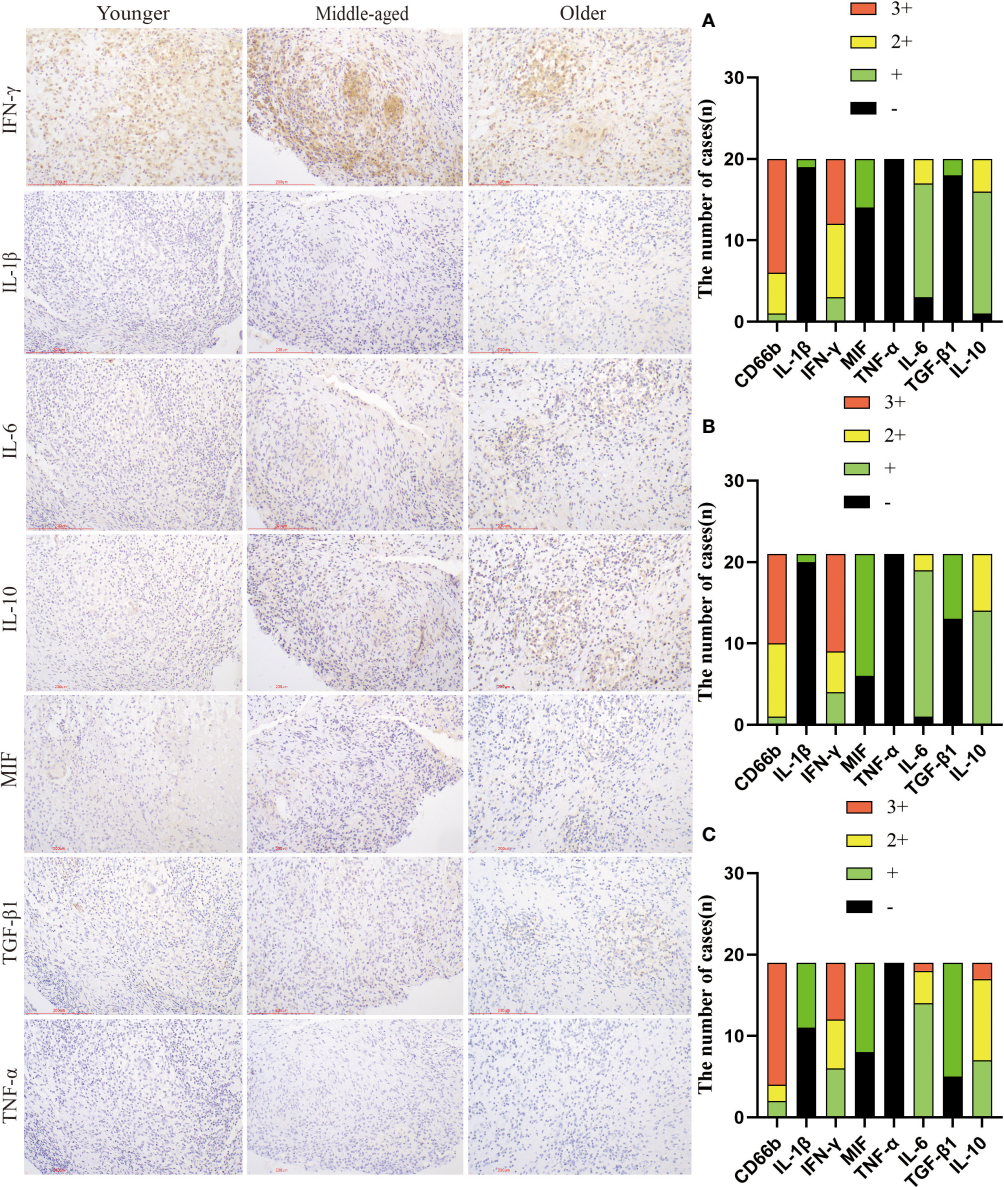
Infiltration and expression of inflammatory factors in the three groups. Immunohistochemical staining was used to label inflammatory factor infiltration. **(A)** Younger group. **(B)** Middle-aged group. **(C)** Older group. Scale bar: 200 μm; Image magnification: immunohistochemical staining (200×).

TNF-α, IFN-γ, IL-1β, IL-6, and MIF are pro-inflammatory molecules that activate immune cells and defend against infection. TGF-β1 and IL-10 are common immunosuppressive molecules that negatively regulate T lymphocytes and other immune cells, and can also antagonize the effects of several interleukins, tumor necrosis factor, and interferon. Immunohistochemistry results showed no statistically significant difference in the expression of TNF-α, IL-6, and IFN-γ between the groups. The positive expression rates of TGF-β1 and IL-1β were significantly higher in the older group than in the younger group. Additionally, the positive expression rate of IL-1β was also significantly higher in the older group than in the middle-aged group. The MIF positive expression rate was significantly higher in the middle-aged group compared to the younger group. The high expression rate of IL-10 was significantly greater in the older group than in the younger group, though the positive expression rate of IL-10 did not differ significantly between the groups. (**Figure 5**, **Table 3**).

**Table 3:** Comparison of inflammatory factor expression.

inflammatory factor	Younger (n=20)			Middle-aged (n=21)			Older (n=19)			*P*
	Negative	Low expression	High expression	Negative	Low expression	High expression	Negative	Low expression	High expression	
CD66b	0	5% (n=1)	95% (n=19)	0	4.76% (n=1)	95.24% (n=20)	0	10.53% (n=2)	89.47% (n=17)	0.681
IL-1β	95% (n=19) ^*^	5% (n=1) ^*^	0	95.24% (n=20) ^#^	4.76% (n=1) ^#^	0	57.89% (n=11)	42.11% (n=8)	0	0.002
IFN-γ	0	15% (n=3)	85% (n=17)	0	19.05% (n=4)	80.95% (n=17)	0	31.58% (n=6)	68.42% (n=13)	0.456
MIF	70% (n=14)	30% (n=6)	0	28.57% (n=6) ^##^	71.43% (n=15) ^##^	0	42.11% (n=8)	57.89% (n=11)	0	0.026
TNF-α	100% (n=20)	0	0	100% (n=21)	0	0	100% (n=19)	0	0	1.000
IL-6	15% (n=3)	70% (n=14)	15% (n=3)	4.762% (n=1)	85.714% (n=18)	9.524% (n=2)	0	73.68% (n=14)	26.32% (n=5)	0.269
TGF-β1	90% (n=18) ^*^	10% (n=2) ^*^	0	61.90% (n=13)	38.10% (n=8)	0	26.32% (n=5)	73.68% (n=14)	0	0.000
IL-10	5% (n=1)	75% (n=15) ^*^	20% (n=4) ^*^	0	66.67% (n=14)	33.33% (n=7)	0	36.84% (n=7)	63.16% (n=12)	0.027

Please note that an asterisk (*) indicates a statistically significant difference between the younger and older groups, a pound sign (#) indicates a statistically significant difference between the middle-aged and older groups, and two-pound signs (##) indicate a statistically significant difference between the middle-aged and younger groups.

## 4 Discussion

Patients with spinal tuberculosis often have comorbid pulmonary tuberculosis, with up to 67% of patients with spinal tuberculosis having a primary lung lesion or a history of tuberculosis reported (Mann et al., 2022). To ensure the accuracy of the study, we decided to exclude patients with pulmonary tuberculosis and those with a history of tuberculosis from our data collection. The study was limited to patients with simple spinal tuberculosis.

Spinal tuberculosis is the most common form of bone and joint tuberculosis, predominantly affecting the vertebrae. The tuberculosis bacterium can destroy the soft tissues between the vertebrae, causing them to fuse and narrowing the intervertebral space. Due to restricted blood flow, the vertebrae are susceptible to bone destruction, necrosis, caseous necrosis, and abscess formation. During the healing process, the body gradually absorbs part of the lesion products, which can lead to the proliferation of fibrous tissue and ultimately result in fibrous and bony healing. Early diagnosis, standardized treatment, and precise treatment significantly improve the prognosis of patients with spinal tuberculosis. Spinal tuberculosis surgery is usually preceded by two to four weeks of anti-tuberculosis treatment to keep the *Mycobacterium tuberculosis* in a quiescent phase. Our research revealed that the older group had a lower percentage of abscess formation compared to the younger and middle-aged groups before receiving anti-tuberculosis treatment. This could be attributed to a stronger immune response and increased infiltration of immune cells in younger patients. The unique structure of the spine makes it challenging to eliminate necrotic material that can cause abscess formation. Interestingly, the postoperative pathologic results showed a higher percentage of abscesses in the older group than in the younger and middle-aged groups, which contradicted the imaging findings. The reason for this phenomenon could be that the older group’s mild immune response might result in micro-abscesses that are challenging to detect through imaging. In addition, younger patients have stronger immunity and better preoperative anti-tuberculosis treatment, and abscesses are more easily cleared. Our findings offer guidelines for preoperative anti-tuberculosis treatment for spinal tuberculosis. For instance, extended preoperative anti-tuberculosis treatment may be appropriate for older patients. The WHO recommends that patients with tuberculosis of the bones or joints should receive nine months of treatment (Shanmuganathan et al., 2023). While 6 months of treatment is reportedly considered adequate, many experts still prefer 12-24 months of treatment (Garg and Somvanshi, 2011). On the other hand, it has been reported that surgical treatment is necessary for patients who have not responded to conservative treatment (with or without bracing or bed rest) after 3-4 weeks, due to pain or neurological deficits (Mak and Cheung, 2013). Persistent infection, progressive destruction of vertebral structures, or ensuing instability and deformity may cause pain. Imaging examination can be used to monitor the effectiveness of anti-tuberculosis treatment for uncertain cases.

Spinal tuberculosis is diagnosed through pathology and typically involves chronic inflammation with caseous necrosis. Our previous study found higher rates of caseous necrosis, acute inflammation, abscess, exudation, and granulation tissue formation in spinal tuberculosis compared to pulmonary tuberculosis (Wu et al., 2023). In this study, none of the pathologic features, except for abscesses, were statistically different between the groups. We examined the number and distribution of various immune cells in spinal tuberculosis lesions. The numbers of T cells, CD4^+^T cells, CD8^+^T cells, macrophages, lymphocytes, plasma cells, and NK cells were not statistically different between the three groups. CD4^+^ and CD8^+^ T cells were detected in HIV-negative spinal tuberculosis tissue sections, along with a population of CD68-positive macrophages in the tissue microenvironment (Danaviah et al., 2013). The number of CD20^+^ B cells was significantly higher in the younger group compared to the middle-aged and older groups. However, the clinical significance and value of this finding are unclear. B cells mediate humoral immune function and support the fight against TB. A decrease in B-cell immune response capacity may reduce the ability to clear *Mycobacterium tuberculosis*. While the function of immune cells in pulmonary tuberculosis has been widely studied, their role in tuberculous intervertebral disc tissue has been scarcely reported (Chen et al., 2022).

We compared the expression of inflammation-related factors and found significantly higher rates of positive TGF-β1 expression and high IL-10 expression in the older group compared to the younger group. TGF-β1 and IL-10 are important molecules that suppress the immune system. Research shows that increased expression of these molecules reduces the body’s defense against pathogens. TGF-β1 is a potent cytokine that significantly diminishes the biological function of macrophages (Yang et al., 2022), and also hinders the ability of TNF-α and IFN-γ to kill *Mycobacterium tuberculosis* (Hirsch et al., 1994). It has been suggested that TGF-β1 could potentially contribute to tissue damage and fibrosis in individuals suffering from TB (Kumar et al., 2016). Hyperactive TGF-β1 was detected in the lung lavage fluid and macrophages of patients with pulmonary tuberculosis (Aung et al., 2000; Bonecini-Almeida et al., 2004). Silencing the TGF-β1 gene along with first-line antituberculosis medications has been reported as a strategy to enhance the efficacy of antituberculosis drugs by promoting *Mycobacterium tuberculosis* clearance (Yang et al., 2022). IL-10 can combine with other inflammatory factors to suppress the body’s immune response, thereby triggering the occurrence and development of TB (Ma et al., 2019). An increase in IL-10 levels appears to aid *Mycobacterium tuberculosis* in surviving within the host body (Cavalcanti et al., 2012). It has been found that the combined production of the immunosuppressants IL-10 and TGF-β in patients with pulmonary tuberculosis may reduce host immunity against *Mycobacterium tuberculosis*, resulting in uncontrolled bacterial replication and causing overt disease (Bonecini-Almeida et al., 2004). Elevated levels of TGF-β1 and IL-10 in the tissues affected by spinal tuberculosis can lower the immune system’s ability to kill *Mycobacterium tuberculosis*. This could be one of the reasons why, after a period of anti-tuberculosis treatment, the proportion of abscesses is higher in older patients as compared to younger patients. IL-1β is a pro-inflammatory cytokine, and several studies have emphasized the importance of strictly regulating IL-1β during TB treatment (Silverio et al., 2021). Elevated levels of IL-1β are associated with tissue necrosis and cavity formation in TB patients (Tsao et al., 2000).

Structural and circulatory specificities result in different drug distribution in the spine for TB treatment. Drugs often fail to maintain effective concentrations in the sclerotic areas of the vertebral body and in the more closed tuberculous lesions, which leads to long-term recurrence in patients with spinal tuberculosis (Yang et al., 2022). Impaired immune function has been reported in elderly patients with pulmonary tuberculosis and the need to assess the immune status of patients to improve treatment outcomes (Liu et al., 2022). Based on our findings, monitoring IL-10 and TGF-β1 levels in older patients with spinal tuberculosis may be useful for evaluating the patient’s immune function and disease progression. It can also aid in making timely adjustments to drug therapy programs and deciding when surgical treatments are necessary.

## 5 Conclusions

The imaging examination before preoperative anti-tuberculosis therapy revealed a lower proportion of spinal tuberculosis abscesses in the older group, while the pathological examination of surgical specimens showed that the proportion of abscesses in the older group was higher than that in the younger and middle-aged groups. The high expression of IL-10 and TGF-β1 in the older group may have reduced their anti-tuberculosis immunity and treatment effectiveness. The timing of surgery and the duration of anti-tuberculosis treatment should vary in patients of different ages.

## Data availability statement

The raw data supporting the conclusions of this article will be made available by the authors, without undue reservation.

## Ethics statement

The studies involving humans were approved by the Ethical Committee of Chinese PLA General Hospital. The studies were conducted in accordance with the local legislation and institutional requirements. The participants provided their written informed consent to participate in this study. Written informed consent was obtained from the individual(s) for the publication of any potentially identifiable images or data included in this article.

## Author contributions

SL: Writing – original draft, Writing – review & editing, Data curation, Formal Analysis, Investigation, Methodology, Resources, Software, Visualization. RW: Resources, Visualization, Writing – review & editing. MF: Investigation, Methodology, Writing – review & editing. HZ: Writing – review & editing. DL: Writing – review & editing. FW: Conceptualization, Writing – review & editing. WC: Conceptualization, Funding acquisition, Project administration, Writing – review & editing.
